# Heart Rate Asymmetry Analysis During Head-Up Tilt Test in Healthy Men

**DOI:** 10.3389/fphys.2021.657902

**Published:** 2021-04-13

**Authors:** Rafał Pawłowski, Katarzyna Buszko, Julia L. Newton, Sławomir Kujawski, Paweł Zalewski

**Affiliations:** ^1^Department of Biostatistics and Theory of Biomedical Systems, Faculty of Pharmacy, Ludwik Rydygier Collegium Medicum in Bydgoszcz, Nicolaus Copernicus University, Toruń, Poland; ^2^Population Health Sciences Institute, The Medical School, Newcastle University, Newcastle upon Tyne, United Kingdom; ^3^Department of Hygiene, Epidemiology, Ergonomics and Postgraduate Education, Faculty of Health Sciences, Ludwik Rydygier Collegium Medicum in Bydgoszcz, Nicolaus Copernicus University, Toruń, Poland

**Keywords:** heart rate asymmetry, head-up tilt test, heart rate variability, Poincaré plot, Guzik’s index, Porta’s index

## Abstract

The purpose of this study is to assess the cardiovascular system response to orthostatic stress in a group of 133 healthy men using heart rate asymmetry (HRA) methods. HRA is a feature of variability in human heart rate which is dependent upon external and internal body conditions. The initial phases of head-up tilt test (HUTT), namely, supine and tilt, were chosen as the external body affecting factors. Various calculation methods of HRA, such as Porta’s index (PI), Guzik’s index (GI), and its variance based components, were used to assess the heart rate variability (HRV) and its asymmetry. We compared 5-min ECG recordings from both supine and tilt phases of HUT test. Short-term HRA was observed in 54.1% of men in supine phase and 65.4% of men in tilt phase. The study revealed significant increase of GI (from 0.50 to 0.52, *p* < 0.001) in the tilt phase as well as significant changes in HRV descriptors between HUTT phases. Our results showed that the variability of human heart rate and its asymmetry are sensitive to orthostatic stress. The study of short-term HRA is a potential additional tool to increase sensitivity in conditions where HUTT is a diagnostic tool, such as vasovagal syncope.

## Introduction

The healthy human heart does not beat regularly. Changes in the dynamics of heartbeat are observed naturally during physical exercise as well as during rest. The main reason for the presence of these changes at rest is respiratory sinus arrhythmia, which is a natural heart rate change occurring during each breathing cycle. Studies exploring the dynamics of intervals between heartbeats have shown a relationship between heart rate variability (HRV) and human health. Low cardiac cycle fluctuations can indicate a range of problems—particularly direct cardiac issues (e.g., reduced HRV after myocardial infarction), as well as other medical problems (e.g., decreased HRV has been noted in those with chronic liver disease or clinical depression) (Carney et al., 2001; Mani et al., 2008).

Research over two decades ago has indicated that HRV is not symmetrical in time. This phenomenon, known as heart rate asymmetry (HRA), occurs when the input of decelerations and accelerations to HRV differs in its participation in short and long-term variability. In short-term variability, input of decelerations is superior to accelerations, while in long-term variability, there is an opposite relationship. This means that the human heart accelerates with longer sequences of smaller differences between RR intervals, and slows down with a shorter series of steps with greater differences. This phenomenon was described in detail by Piskorski and Guzik (2011).

Due to the occurrence of HRA in the majority of the healthy human population, it has been postulated that the cardiovascular response to external body conditions or illnesses may manifest as a modification in the asymmetry in human heart beat variability. Research has shown that conditions that can affect HRV and its asymmetry are, among others, gastric cancer, mental stress, controlled breathing, or walking (Wang et al., 2013, 2018; Parvaneh et al., 2015; Shi et al., 2019). Naturally, changes in body position affect HRA particularly when it has rapid course.

Postural orthostatic disturbances are primary manifestation of autonomic failure due to many independent factors; therefore, the cardiovascular effectors’ response to head-up provocation is particularly important. Orthostatic response is influenced by a number of factors, but beat-to-beat control of blood pressure is strictly depended upon the complex sympathetic efferent pathways. In this particular situation, the blood pressure may fall rapidly and progressively, and the rate of recovery depends on the ability of activation of spinal sympathetic reflexes and humoral compensatory mechanisms. These complex reactions affect directly HRV on each phase; therefore, methods of quantifying the heart rate changes are so important in process of dysautonomia diagnosis. Advanced medical devices let to analyze non-invasively the heart rate in beat-to-beat mode, but in some specific conditions, it is difficult to distinguish specific phases of head-up response. Among many available linear methods, HRA as a non-linear method brings a novel and validated approach to the HRV assessment.

Previous attempts have been made to estimate the change in HRA during orthostasis in past. De Maria et al. (2019) investigated HRA at rest in supine position and during active standing. Casali et al. (2008) showed that the cardiovascular response to the vertical position in the form of changes in HRA is different for tilt and standing (the asymmetry between accelerations and decelerations in heart rate appears to be more meaningful in tilt that in standing). (Porta et al. (2008) carried out HRA analysis during HUTT on a group of healthy adults with no gender distinction. The study conducted by Reulecke et al. (2016) revealed significant differences in irreversibility indices behavior (including HRA index) during orthostatic stress (70°) between genders.

In this current study, we set out to assess cardiovascular system response during a controlled hemodynamic response to tilt in healthy men. We used a head-up tilt test (HUTT) as a standardized orthostatic stress and aimed to study the impact of various external and internal factors on HRA. The HUT tests analyzed in this paper were performed with a Task Force Monitor (TFM) device (Schwalm, 2007; CNSystems, 2020). The ECG signals recorded during the tests were used in our analysis.

The aim of our study was to assess the cardiovascular response in men to the initial phases of passive tilt test with focus on using HRA methods.

## Materials and Methods

A single-center, retrospective analysis was conducted on a group of 133 healthy males (34 ± 9 years old). The youngest man was 23 years old, while the oldest was 66. Mean BMI 25.9 ± 3.2 ranged from 18.8 to 38.1. The passive tilt test was performed for each individual in the Department of Hygiene, Epidemiology, Ergonomics and Postgraduate Education in Collegium Medicum in Bydgoszcz. Participants were invited to complete a written informed consent prior to the tilt test and provided written agreement for the use of the collected data for scientific purposes. The study was conducted in accordance with the principles included in the Declaration of Helsinki and was approved by the Ethics Committee, Ludwik Rydygier Memorial Collegium Medicum in Bydgoszcz, Nicolaus Copernicus University, Toruń.

The HUTT is used to diagnose hemodynamic and autonomic nervous system dysfunction. The main principle of the HUTT involves the participant lying on a table that can tilt to different angles in a short time, the angle, the time in supine position and tilt are precisely defined. The subject’s electrocardiograph (ECG) and blood pressure are monitored during the test. Depending on the equipment used for the HUTT, non-invasive cardiovascular parameters can also be measured using impedance cardiography (ICG). In our project, the HUT tests were performed using the TFM system device (CNSystem, Graz, Austria). This system is composed of two main parts: a lift table with a footboard and abdominal straps and devices for continuous monitoring and recording of ECG and blood pressure. The biosignals are recorded on a beat-to-beat basis. The TFM device is equipped with high-resolution two-channel ECG with a sampling frequency of 1000 Hz. The algorithms extracting the RR intervals from ECG are implemented in the system.

**FIGURE 1:**
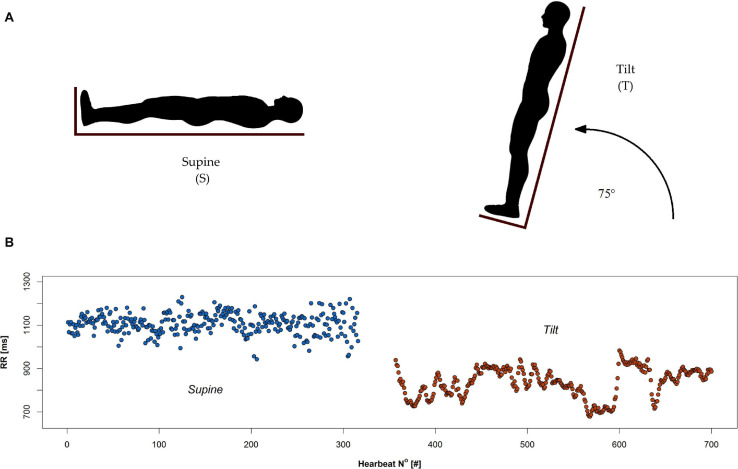
Head up tilt test: **(A)** schematic illustration of supine (S) and tilt (T) phases, and **(B)** an example of RR series recorded during the test.

The protocol of the study was as follows: the participants arrived at the unit having fasted prior to the test. Each examination was conducted in the morning in a quiet room with dimmed lighting. All volunteers were healthy men with current medical tests indicating the absence of disease (including routine laboratory tests). Exclusion criteria consisted of factors that could possibly modify HRV: shift work, caffeine, alcohol, drugs dependence, participation in sports at competitive level, alcohol consumption within 12 h before the test, receiving any medication/supplements during the examination, and potential disorders of the cardiovascular system observed during the test. All potential study participants were questioned about their sleep quality, life habits, and health state. Pre-test health state assessment of subjects included: basic clinical (physical) examination, evaluation of blood test, and any of available medical tests/examination; enrolment was done by consultant and clinical physiologist. At the beginning of the test, the man remained in the supine position for 10 min until signals were stabilized and afterward he was tilted to 75° in 19 s (Figure 1). The passive upright tilt duration lasted 5–6 min.

During all phases of the test, the ECG signal was recorded. Figure 1 demonstrates two phases of tilt test: supine and tilt (A) with an example of the RR interval series extracted from ECG signal during the test (B).

We analyzed the ECG signal recorded during the 5-min period of rest in the supine position (S), and afterward during the 5 min period of tilt position (T). All calculations were conducted on normal-to-normal RR cycles, i.e., having their origin in the sinus node. The non-sinus origin heartbeat intervals (i.e., supraventricular and ventricular ones) from the data were excluded by filtering the data using a computerized filter (Piskorski and Guzik, 2005). Subjects with more than 5% of the RR pairs removed by a quotient filter were excluded from the study group.

We analyzed the RR intervals using HRA methods (Piskorski and Guzik, 2011; Piskorski et al., 2019). Initial analysis involved investigation of all 5-min recordings in the supine and tilt position. In order to estimate the dependence between age and analyzed HRA parameters, we divided study group into three age-based subgroups: ≤ 30 years old (group 1), 31–40 years old (group 2), and over 40 years old (group 3). We carried out the HRA analysis between the age groups to detect the possibility of age effect on our results. Second, all 5-min recordings were cut into 150-beat RR segments (windows). The windows were overlapping each other and sliding through the 5-min sequence with a step of 1 heartbeat.

Moreover, using ECG recordings, we performed power spectral analysis. We determined the components of spectral analysis: low frequency (LF) power (0.04–0.15 Hz), high frequency (HF) power (0.15–0.40 Hz), and their ratio (LF/HF). The adaptive auto-regressive (AAR) model was used to compute the time-varying spectral estimation (Bianchi et al., 1997). The components were examined in supine and tilt due to evaluation of both sympathetic and parasympathetic influences (LF), modulation of vagal tone (HF), and the balance between sympathetic and parasympathetic tones (LF/HF).

The analyses of HRA based on Poincaré plot (PP) descriptors and monotonic series of RR intervals occurrence have been performed with the *hrvhra* package (version 0.1.0; author: Jaroslaw Piskorski^1^).

All statistical analyses and all figures were obtained with R (version 3.6.2^2^). Descriptive statistics included: means and standard deviation, median, first and third quartile, and interquartile range. All statistical calculation has been performed on significance level α = 0.05. The normality of data distribution was verified with Shapiro–Wilk test. The results obtained for supine and tilt positions were compared using paired *t*-test. When comparing the groups, the Mann–Whitney test has been performed. In order to perform multi-group comparison, Kruskal–Wallis test with *post hoc* analysis was carried out. Differences in proportions were established with McNemar’s test.

## HRV and HRA

**FIGURE 2:**
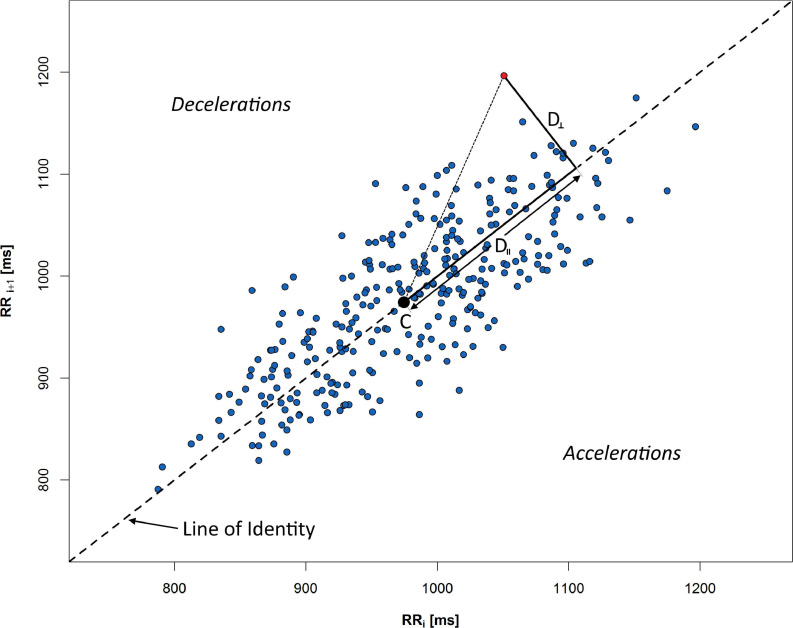
Poincaré plot of 5 min RR sequence recorded during rest in supine. Distances from selected red point to the centroid C are measured along the LI (D_∥_), and perpendicular to the LI (D_⊥_). All points above LI respond to the decelerations of human heart rate, whereas points under the line indicate accelerations (consecutive shortening of RR time distance).

Mathematical methods of HRA analysis sensitive to a difference between short- and long-term variability were considered. The geometric method of HRV measurement is PP. In this kind of graph, each RR interval is plotted against the consecutive one (see Figure 2). For a healthy person, this graph takes the shape of an asymmetrical ellipse-like figure. The crucial elements of the chart are: the line of identity (LI), where RR_i_ = RR_i__+__1_, and the centroid of the cloud of all points on the graph. Based on the analysis of the dispersion of points on the chart, it is possible to determine metrics of short- and long-term HRV and HRA. The boundary between long- and short-term variability is not clearly established; however, it is possible to define long- and short-term components of PP-based HRV and HRA descriptors. RR interval series may be divided into monotonic runs in which RR_i_ > RR_i__+__1_ for accelerating series and RR_i_ < RR_i__+__1_ for decelerating ones. In the approach presented in this work, the monotonic RR interval series of length equal to one correspond to short-term variability only. The longer the sequence, the bigger its contribution into long-term variability (Brennan et al., 2001).

An example of PP with crucial elements for HRA description is presented in Figure 2: C is a centroid of ellipse-like figure made of PP points, D_⊥_ is a distance from a given point to LI, and D_∥_ is a distance measured along LI. The graph is divided into accelerations and decelerations sections—depending on relative position to the LI.

The variance-based RR series descriptors allow us to distinguish the contribution of RR series into long- and short-term HRV. *Guzik’s index* (GI) is an index which allows the calculation of decelerations contribution into short-term HRV:

(1) $$\text{GI}=\frac{{\mathrm{SD1d}}^{2}}{{\mathrm{SD1}}^{2}}$$

where

(2) SD1d2=1n⁢∑i=1nD⊥2i

is the mean of the squared perpendicular distances D_⊥__i_ (Figure 2) from the LI of all n_+_ points on the PP which are located above the identity line and, accordingly

(3) SD12=1n⁢∑i=1nD⊥2i

is the mean of corresponding distances of n points located both below and above the LI (Guzik et al., 2006). Short-term HRA is understood as a situation, when GI > 0.5.

Long-term variability alternative for GI is the contribution of decelerations into long-term HRV:

(4) $${\text{GI}}_{\text{LT}}=\frac{{\mathrm{SD2d}}^{2}}{{\mathrm{SD2}}^{2}}$$

where SD2d and SD2 are obtained analogously to (3) and (4) with the difference that the distances D_∥_ are measured in parallel to LI (as projections of the distance onto LI—see Figure 2). SD1 is a measure of the distribution of points crosswise to LI and is a measure of short-term HRV, while SD2 measures the distribution along LI and is a measure of long-term HRV. Long-term HRA occurrence is understood as a situation, when GI_LT_ < 0.5 (Piskorski and Guzik, 2011).

Another metric based on PP is *Porta’s index* (PI). It can be calculated as

(5) $$\text{PI}=\frac{{\text{n}}_{-}}{\text{n}}\cdot 100$$

where n_–_ stands for the number of points below the identity line (accelerations) and n is the total number of points which do not lie on the LI (Porta et al., 2008).

Mathematical methods of HRA calculations are constantly being developed. There are attempts to redefine currently used descriptors (e.g., redefined GI in Karmakar et al., 2012) and to create new ones, as slope index, area index, or SKG index (Karmakar et al., 2015a; Yan et al., 2017; Kramaric et al., 2019).

Mean RR interval, SD1, SD2, GI, GI_LT_, and PI were calculated within 5 min RR series registered in tilt and supine. Afterward, subjects with short-term HRA occurrence (GI > 0.5) in whole 5-min recordings have been selected for analysis in sliding *windows*. RR recording has been cut into sections (windows) of length 150 RR intervals. Each window was prepared in the following way: first one is a series consisting of the first 150 RR intervals: {RR_1_, RR_2_, …, RR_150_}, second one—{RR_2_, RR_3_, …, RR_151_}, etc., until {RR_k–149_, RR_k–148_, …, RR_k_}, where k is a number of all intervals in 5-min series. GI was calculated for each window, and the occurrence of asymmetry has been established. The percentage of windows with short-term HRA occurrence (Π) was calculated.

The ECG recording can be divided into subsequences of ascending RR intervals (i.e., heart rate decelerations), descending ones (accelerations), and constant ones. The monotonic strings have been marked as *dec.* and *acc.*, respectively. The *analysis of the length of monotonic RR interval series* has been performed to estimate the changes of the length during HUTT.

## Results

### Heart Rate Variability

The results of the analyses presented in this section were obtained using the RR intervals recorded during the HUT tests described in the previous section.

**FIGURE 3:**
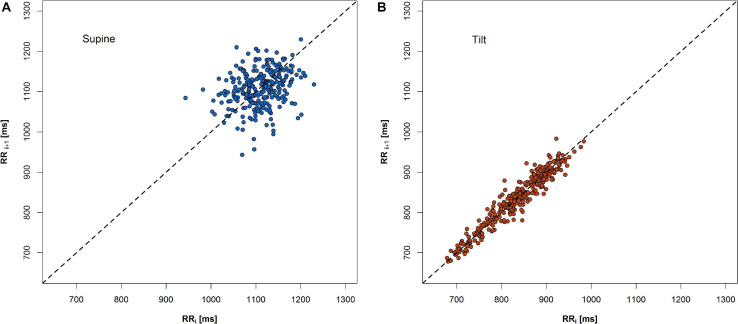
An example of change of PP shape during 5-min recordings in two phases of HUTT: **(A)** supine and **(B)** tilt.

A change of PP shape as a result of an HRV variation during HUTT has been shown in Figure 3.

**FIGURE 4:**
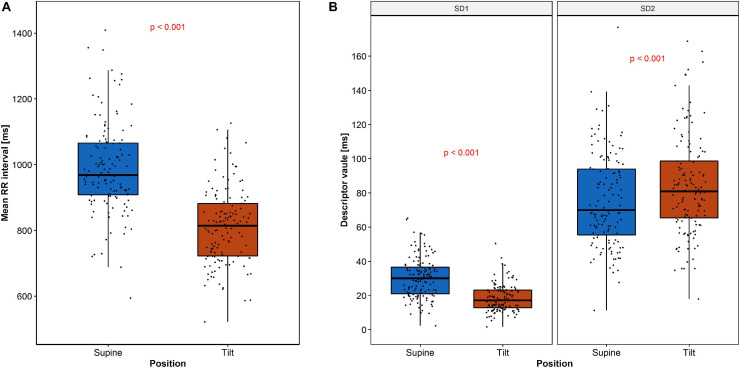
Changes of RR intervals and HRV descriptors during HUTT: **(A)** mean RR intervals and **(B)** SD1 and SD2. The dots represent the data.

The expected heart rate increase in response to the tilt manifests in PP as a shift of points along the LI to the lower values in the tilt (Figure 3B) compared to the supine (Figure 3A). Another observation is that the PP shape narrowed and elongated in parallel to the LI. Both heart rate increase and a reduction of SD1 parameter with simultaneous growth of SD2 parameter have been observed (Figure 4).

The descriptive statistics of RR interval length and both SD1 and SD2 parameters are summarized in Table 1.

**TABLE 1:** RR intervals and heart rate variability descriptors measured during 5 min in supine position (S) and 5 min in tilt (T).

	Min	Max	Q1	Median	Q3	IQR	Mean	SD
***RR intervals***								
RR (S) [ms]	562.00	1750.00	882.08	961.04	1054.04	171.96	970.78	146.62
RR (T) [ms]	323.38	1367.33	699.33	787.04	874.08	174.75	795.12	128.27
***Short-term HRV***								
SD1 (S) [ms]	2.29	65.35	20.96	29.97	36.54	15.59	30.24	11.80
SD1 (T) [ms]	1.65	50.44	12.85	17.13	23.13	10.28	18.36	7.79
***Long-term HRV***								
SD2 (S) [ms]	11.25	176.88	55.33	69.96	93.94	38.61	75.20	26.79
SD2 (T) [ms]	17.96	168.75	65.33	80.86	98.61	33.28	83.88	28.74

### Spectral Analysis of Heart Rate Variability

The power spectral analysis revealed significant difference (*p* = 0.045) for LF power and highly significant difference (*p* < 0.0001) for both HF power and LF/HF power ratio. The descriptive statistics of LF, HF, and LF/HF parameters are summarized in Table 2.

**TABLE 2:** Power spectral analysis parameters in supine (S) and tilt (T) stages of HUTT.

	Min	Max	Q1	Median	Q3	IQR	Mean	SD
***Low Frequency***								
LF (S)	5.565	4331.301	324.901	584.471	1235.605	910.704	913.207	782.438
LF (T)	6.474	2762.102	357.151	569.141	1094.279	737.128	814.499	624.007
***High Frequency***								
HF (S)	1.818	4237.962	224.738	454.381	904.579	679.841	701.784	713.614
HF (T)	0.987	2056.650	94.815	181.115	437.164	342.349	297.475	306.446
***Ratio***								
LF/HF (S)	0.145	15.963	1.013	1.469	2.275	1.262	1.973	1.813
LF/HF (T)	0.899	28.114	2.420	3.688	5.722	3.302	4.846	3.911

### Heart Rate Asymmetry

Short-term HRA (i.e., GI > 0.5) was observed in 72 out of 133 cases (54.1 %) during rest and in 87 out of 133 cases (65.4 %) during tilt. No significant difference between proportions of short-term HRA occurrence was observed (*p* = 0.064).

Guzik’s index and PI have been calculated for all participants both in supine and in tilt position. The results are summarized in Table 3.

**TABLE 3:** Descriptive statistics of rate asymmetry descriptors: Guzik’s index and Porta’s index dispersion among participants during HUTT in supine (S) and tilt (T) positions.

	Min	Max	Q1	Median	Q3	IQR	Mean	SD
***Guzik’s Index***								
GI (S)	0.3396	0.6217	0.4823	0.5049	0.5251	0.0428	0.5001	0.0443
GI (T)	0.3619	0.6257	0.4864	0.5225	0.5596	0.0732	0.5208	0.0515
***Porta’s Index***								
PI (S)	44.61	62.73	49.45	51.43	53.33	3.88	51.71	3.32
PI (T)	42.93	62.12	49.73	51.93	54.52	4.79	52.05	3.47
***Long-term Guzik’s Index***								
GI_LT_ (S)	0.3180	0.5726	0.4230	0.4621	0.5037	0.0808	0.4622	0.0567
GI_LT_ (T)	0.3103	0.6115	0.4286	0.4671	0.5011	0.0725	0.4638	0.0519

**FIGURE 5:**
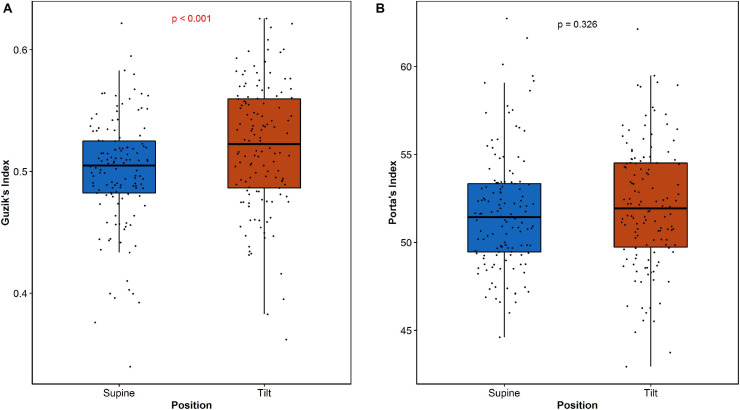
The comparisons of indexes GI **(A)** and PI **(B)** in two HUTT phases (supine and tilt).

The differences between short-term asymmetry descriptors calculated in supine and tilt are statistically significant for GI (*p* < 0.001) and not significant for PI (*p* = 0.326). The comparison of these parameters is presented in Figure 5.

GI_LT_ analysis led to observation of long-term HRA occurrence in 98 participants (73.7%) in both tilt and supine positions. There has been no significant changes between supine and tilt for GI_LT_ values (*p* = 0.766).

**FIGURE 6:**
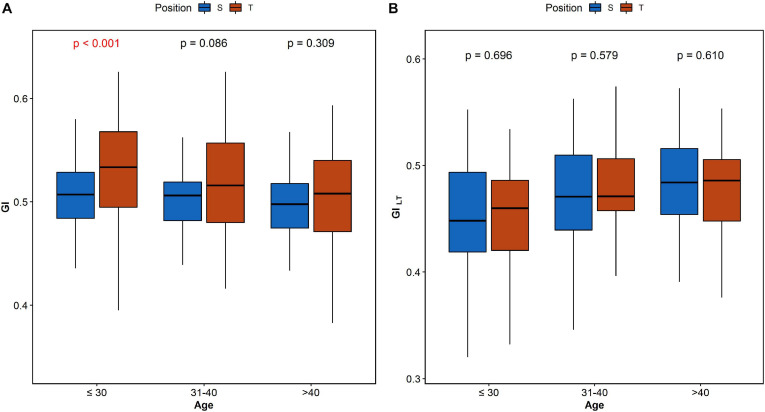
Comparison of GI **(A)** and GI_LT_ **(B)** between supine and tilt in each age group.

The analysis of HRA between the age groups reveals significant difference of GI_LT_ between age categories in both supine and tilt. The results are summarized in Table 4. *Post hoc* analysis revealed significant difference (*p* = 0.036) between age groups 1 and 3 (Table 4B). Furthermore, we compared each parameter between supine and tilt within each age group. No significant changes in PI between S and T in any of groups have been observed. Comparison of GI and GI_LT_ within age groups is presented in Figure 6.

**TABLE 4:** Age analysis of HRA indices (GI, GI_LT_, and PI): age categorization (**A**); analysis of each parameter between age groups in supine and tilt (**B**); and *post hoc* comparison of GI_LT_ in supine and tilt (**C**).

(A)	Age category	Age (min = 23; max = 66)	Number of participants
	1	≤30	67
	2	31–40	38
	3	>40	28

(**B**)		**Kruskal–Wallis *p***	
	**Index**	**S**	**T**

	GI	*p* = 0.606	*p* = 0.077
	GI_LT_	*p* = 0.024	*p* = 0.033
	PI	*p* = 0.105	*p* = 0.163

(**C**)		**GI_LT_*post hoc***	
	**Age cat.**	**S**	**T**

	1–2	0.205	0.087
	2–3	1.000	1.000
	3–1	0.036	0.117

### Short-Term HRA Analysis in Sliding Windows

**FIGURE 7:**
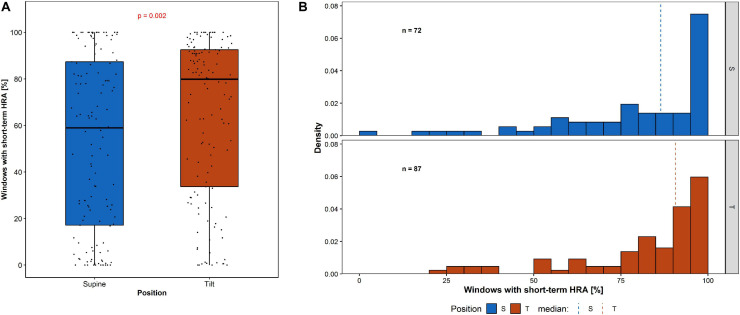
Percentage of short-term HRA occurrence in 150-beat windows: **(A)** among all participants and **(B)** the distribution of windows with short-term HRA occurrence among participants with GI > 0.5 in whole 5-min recording.

The analysis of percentage of short-term HRA occurrence in 150-beat windows (Π) has been performed and compared in supine and tilt for all participants (Figure 7A), and for the selected group of subjects with short-term HRA occurrence in whole 5-min recordings (Figure 7B).

The percentage of windows with short-term HRA occurrence of subjects in supine compared to the same participants in tilt is statistically significant (*p* = 0.002). The Π in the group of subjects with GI > 0.5 in 5-min recording in supine (72 participants) compared with the Π in the group of subjects with GI > 0.5 in 5-min recording in tilt (87 participants) is not statistically significant (*p* = 0.801). The results of short-term HRA observations in windows are summarized in Table 5.

**TABLE 5:** Percentage occurrence of HRA in 150-beat sliding windows for all 133 participants, 72 with short-term HRA occurrence registered in 5-min recording in supine and 87 with short-term HRA occurrence registered in 5-min recording in tilt.

	Min	Max	Q1	Median	Q3	IQR	Mean	SD
***All participants:***								
Π (*n* = 133) (S)	0.00	100.00	17.14	58.93	87.35	70.21	52.35	37.31
Π (*n* = 133) (T)	0.00	100.00	33.68	79.84	92.55	58.86	64.52	33.75
***Selected participants:***								
Π (*n* = 72)^1^ (S)	2.50	100.00	67.05	86.43	98.87	31.82	79.57	22.96
Π (*n* = 87)^1^ (T)	21.99	100.00	78.10	90.65	97.09	18.99	82.23	19.89

*^1^Calculations carried out among subjects with short-term HRA occurrence in 5-min recording in supine (*n* = 72) and tilt (*n* = 87).*

Analysis of Π performed for long-term asymmetry in group of all participants shows no significant difference between supine and tilt (*p* = 0.266).

### Monotonicity of RR Sequences

Monotonic RR sequence is a part of recording in which RR_i_ < RR_i__+__1_ or RR_i_ > RR_i__+__1_ for all intervals (constant strings RR_i_ = RR_i__+__1_ have been omitted in research due to the negligible number of occurrences). The length of ascending RR sequences (heart rate decelerations) and descending ones (accelerations) varies from one to over a dozen. Descriptive statistics for ascending (*dec.*) and descending (*acc.*) RR interval strings length is presented in Table 6.

**TABLE 6:** Descriptive statistics of length of monotonic RR sequences extracted from 5-min ECG recordings in supine and tilt positions.

	Min	Max	Q1	Median	Q3	IQR	Mean	SD
*dec.* (S)	1	9	1	2	2	1	1.97	1.04
*dec.* (T)	1	17	1	2	3	2	2.57	1.72
*acc.* (S)	1	12	1	2	3	2	2.09	1.28
*acc.* (T)	1	19	1	2	4	3	2.81	2.17

**FIGURE 8:**
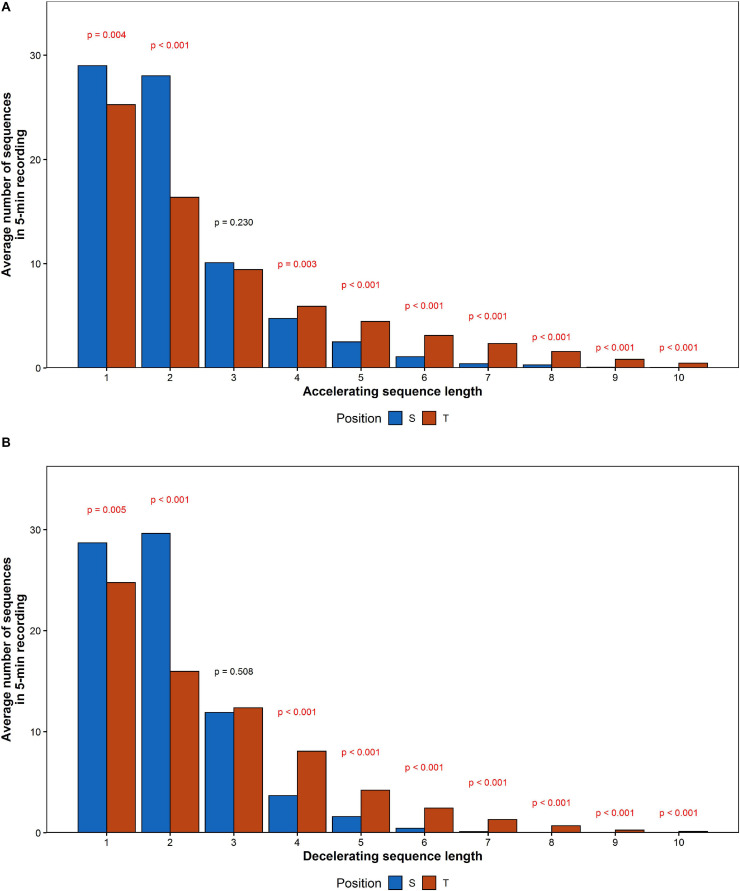
The comparison of mean number of **(A)** accelerating and **(B)** decelerating monotonic RR series recorded during the HUTT.

The average RR sequence length increased by 0.72 for accelerations and 0.60 for decelerations. The comparison between mean values calculated for each of acceleration and deceleration sequences length has been shown in Figure 8.

The number of series shorter than three intervals decreased, while series longer than three increased their contribution to HRV. To determine the impact of elongation of individual series on HRA, additional comparison of number of monotonic sequence of the same lengths has been performed between accelerations and decelerations in supine and tilt positions. The results are presented in Table 7. Due to the availability of insufficient data, we were not able to conduct the comparison between series longer than eight subsequent RR intervals in supine and 10 intervals in tilt.

**TABLE 7:** Comparison of average number of deceleration and acceleration series in supine and tilt. Monotonic series of no more than eight consecutive heartbeats has been compared in supine, and no more than 10 heartbeats in tilt.

RR sequence length	Median (mean)					
	Supine			Tilt		
	*dec.*	*acc.*	*p*-value	*dec.*	*acc.*	*p*-value
1	25 (28.70)	27 (29.00)	0.649	23 (24.76)	23 (25.27)	0.331
2	31 (29.63)	28 (28.03)	0.026	14 (15.97)	15 (16.38)	0.424
3	12 (11.90)	10 (10.10)	0.001	12 (12.37)	9 (9.44)	0.001
4	3 (3.68)	4 (4.74)	0.001	7 (8.07)	6 (5.92)	0.001
5	1 (1.59)	2 (2.50)	0.001	4 (4.21)	4 (4.45)	0.438
6	0 (0.45)	0 (1.08)	0.001	2 (2.44)	3 (3.12)	0.007
7	0 (0.12)	0 (0.40)	0.003	1 (1.31)	2 (2.35)	0.001
8	0 (0.03)	0 (0.29)	0.003	0 (0.69)	1 (1.59)	0.001
9	—	—	—^1^	0 (0.26)	0 (0.81)	0.001
10	—	—	—^1^	0 (0.13)	0 (0.47)	0.001

*^1^Insufficient data.*

The number of registered accelerations in supine exceeds the number of decelerations for all series longer than three intervals. Analogous inequality in tilt has been observed only for series longer than five intervals. There is a noticeable disappearance of the difference between accelerations and deceleration with a length equal to 2, while the contribution of decelerations into short-term HRV during tilt increased (higher GI).

## Discussion

Changes in HRV and HRA descriptors during HUTT have been observed in this study in the majority of healthy men. Increase in the heart rate manifests as a shift of the PP (i.e., RR interval shortening). Change in the PP shape during tilt test indicates the increase of long-term variability contribution to total HRV with simultaneous decrease in the short-term variability input. The analysis of SD1 and SD2 descriptors as well as the analysis of length of monotonic RR series confirms those observations.

Increased sympathetic activity leads to changes in RR through the release of epinephrine and norepinephrine, which activate *β*-adrenergic receptors, leading to an increase in the cardiac Ca^2+^ concentration and the hyperpolarizing-activated current, which in turn leads to acceleration of the slow diastolic depolarization and a decrease of RR interval. On the other hand, sympathetic activity directly influences ventricular repolarization by influencing the Ca^2+^ current of the L-channel and slow delayed rectifier potassium current in ventricular myocytes, which leads to decrease of the repolarization and increase its heterogeneity (Camm et al., 1996). Sympathetic branch seems to be not solely responsible for observed accelerations in HRA (Piskorski and Guzik, 2012). Under physiological conditions, heart rate in rest is under influence of parasympathetic branch, and blockade of its activity leads to slight increase of HR (from 90 to 120 beats per minute) (Hainsworth, 2004). Factors beyond direct action of autonomic nervous system influence on decelerations and accelerations (Piskorski and Guzik, 2012) such as the mechanical effects of intrathoracic pressure fluctuation related to inspiration and expiration (Klintworth et al., 2012), impact of hormones, and O_2_, electrolytes, H^+^ as well as asymmetrical characteristic of the conduction of depolarization wave between atria and ventricles (Camm et al., 1996; Berntson et al., 1997; Guzik et al., 2011). Moreover, five-irreversible dynamics (Casali et al., 2008) as well as time irreversibility of beat-to-beat HRV and BPV were changed in response to orthostatic stress (Chladekova et al., 2012). De Maria et al. (2019) have suggested that HRA is determined by cardiac baroreflex hysteresis. Piskorski and Guzik (2012) suggest that among all factors, presumably autonomic nervous system activity plays the main role in biological underlying mechanism of asymmetry. Moreover, Porta et al. (2008) have suggested a role of autonomic nervous system in temporal asymmetries of short-term heart period variability. However, physiological background of asymmetry should be examined in further studies.

Sundkvist et al. dissected the heart rate response to passive tilting by measuring the acceleration index (the shortest RR interval after standing minus baseline RR interval all divided by the baseline RR interval) and the brake index (the longest RR interval after standing minus the shortest RR interval all divided by the baseline RR interval). They suggested that the acceleration index provides a measure of baroreceptor-mediated vagal response to the sympathetic nervous system-mediated increase in peripheral resistance (Sundkvist et al., 1980). Other authors suggest that the RR interval elongation does not occur after passive tilting. In summary, the knowledge of HRA physiological background is under constant development. Therefore, further research in this topic is highly needed.

Using HRV modeling calculated in time and frequency domain is widely recommended in many clinical protocols. Quantitative HRV analysis is a very well documented tool describing the cardiovascular response in both short- and long-term regulation. Short-term spectral analyses of HRV are a reliable clinical tool with high reproducibility. Rapid changes in response to active or passive tilting have been developed as a clinical approach in syncopy assessment.

Changes of specific HRV spectral bands reflect both physiology and pathophysiology upon orthostatic stress. Porta et al. (2007) showed that spectral density indices are correlated with inclination of the table during HUTT. The power spectral analysis results in our work show significant difference between S and T for all tested parameters. Changes of HRV spectral density were observed in early phase of stabilization and prolonged orthostatic stress. Observed progressive changes reflect an increase in heart rate due to blood volume changes.

The GI difference between rest and tilt in our research is statistically significant, while GI_LT_ is not significant. The PI difference between supine and tilt is not significant. This result suggests that PI may not be sensitive to the distinction between long- and short-term HRA. Wang et al. (2018) showed significant reduction of PI during low-intensity exercise—5 km/h walking—while changes in GI remained insignificant. Thus, our studies suggest that HUTT impacts upon the increase of heart rate in a different way than physical exercise. HRV dynamics during acute bout of physical exercise shows curvilinear decay as a function of exercise intensity, which seems to be dependent on HR dynamics (Michael et al., 2017). HRV measurements related to parasympathetic cardiac activity usually reach a near zero minimum at moderate intensity of physical exercise. Occasionally, these rates are observed to increase slightly as exercise intensity increases to a maximum. What seems interesting, response of frequency domain ratio and normalized measures as indicators of sympathetic activity or “sympatho-vagal balance” during physical exercise are inconsistent with supposed mechanism based on progressive parasympathetic withdrawal and sympathetic activation (White and Raven, 2014).

The analysis of GI is consistent with the results of the previous researches described in De Maria et al. (2019) although PI in our study remained unchanged. Possible difference may arise due to different way of sympathetic activation between passive and active standing. In Porta et al. (2008), the relationship between degree of tilt during graded head-up tilt and HRA was tested on smaller group (17 participants) consisting of men and women. It revealed significant change in HRA indices between supine and tilt only for particular inclination of the table: 75° for GI and 90° tilt for PI. Thus, unchanged PI in our study may appear as a result of lower table inclination as well as study group consisting of only male participants.

The analysis of HRA indices between age groups showed significant difference in GI_LT_ between subjects under 30 and over 40 years old. Further comparison shows that such a dependency has no impact on differences between supine and tilt of GI_LT_ (Figure 6B). The result of GI comparison between supine and tilt within age groups (Figure 6A) indicates an impact of age on GI despite the insignificant difference in GI values in supine and tilt while taking into account the age groups (Table 4B). In De Maria et al. (2019), correlation has been revealed of GI on age at rest and during active standing.

As a result of stimulation of the cervical nerve in humans, Borst and Karemaker found changes in the PP interval 0.5–0.6 s, and changes in the AV interval 1 s after initialization of stimulation. With fixed-rate atrial stimulation, the decrease in diastolic blood pressure began after 2–3 s. Moreover, after stimulation cessation, recovery was of the same delay; therefore, no asymmetry was detected. The authors also concluded that the heart rate response is determined by a reflex drop in blood pressure, and not by the primary stimulus itself. Moreover, difference in delay between effects of vagal compared to sympathetic activity changes was noted. Carotid sinus stimulation induces changes in vagal activity with a delay of approximately 100 ms, while sympathetic activity changes after approximately 150–300 ms (Karemaker and Borst, 1983). Klintworth et al. (2012) propose that sympathovagal interactions are the main, although not exclusive determinants of HRV and HRA. If the output of this interaction is unable to affect the heart rate at exactly the same time, it can lead to asymmetry in accelerating and slowing down the heart rate.

Piskorski and Guzik (2011) found that the acceleration of the heart rate compared to its deceleration has a greater effect on the long-term HRV (GI_LT_ < 0.5) in people in the supine position. In the course of our research, we observed GI_LT_ < 0.5 in 73.7% of participants in supine. Guzik et al. (2006) reported clearly visible short-term asymmetry in 82% of their study group during 5-min rest in a recumbent position and similar results were proclaimed afterward also for longer (30 min) recordings (Piskorski and Guzik, 2007, 2011). Our estimation carried out with the same calculation method for subjects in supine yielded 54% of participants showing HRA. This dissimilarity inspired us to conduct sliding window analysis.

In Yeragani et al. (2000) and (El-Hamad et al. (2015, 2019), mean RR and its variance decreased in response to tilt; however, no distinction was made between long- and short-term HRV. El-Hamad et al. (2019) noted that repolarization variability was independent of HR during HUTT. The mean values of repolarization features decreased with the tilt, while variance of these indicators noted an increase.

In previous research (Piskorski et al., 2019), attempts to assess the ratio of windows with asymmetric HRV in the entire recording were conducted for disjoint jumping windows (i.e., windows did not overlap each other). In our investigations, we divided the recording into overlapping and sliding windows. The analysis of Π in the group of selected subjects has been performed due to the possibility that for the GI nearly equal to 0.5, the number of PP points responsible for occurrence of HRA may be inconsiderable. Our study revealed cases with up to 97.5% of windows with no short-term HRA occurrence in recordings for which the occurrence of short-term HRA has been found. We suggest that future work should focus on investigations of the influence of individual groups of PP points on different HRA metric methods.

During our research, an extension of monotonic RR interval sequence length was observed. The average amount of short series (length < 3) decreased during HUTT, whereas the quantity of long series (length > 3) increased in the tilt position. A lack of difference in long-term HRA occurrence indicates symmetrical length extension of both deceleration and acceleration series after tilt. The fact that the increase of average RR sequence length for accelerations is greater than average length increase of decelerations and the disappearance of the difference between accelerations and deceleration with a length equal to two during HUTT stands in apparent opposition to an increase in the contribution of decelerations to short-term HRV (i.e., GI increase). The likely explanation for this phenomenon is the increase in differences between consecutive heartbeats which is not noticeable in RR series analysis. Thus, decomposition of ECG into monotonic series does not describe the short-term HRV or might need a different insight.

Several studies have examined HRA clinical application in neonates. Index of HRA seems to be a promising tool in neonatal stress examination (Kramaric et al., 2019). Induction of stress led to change in GI in neonatal. Karmakar et al. observed no significant relationship between GI and gestation age. However, significant relationship between PI and gestation age was observed. Authors conclude that development of autonomic nervous system leads to higher fetal HRA and that gestation age is stronger related to asymmetricities of number of accelerations/decelerations in comparison to its magnitude (Karmakar et al., 2015b). Sample asymmetry analysis was shown do detect abnormal heart rate characteristics that occurred before sepsis and systemic inflammatory response syndrome development in neonatal sample (Kovatchev et al., 2003).

Heart rate asymmetry was also applied in adult patients samples. Measure of asymmetry was shown to be diminished in patients with diabetes type 1 (Guzik et al., 2010). In addition, in severe obstructive sleep apnea patients, higher quantity of longer monotonic RR sequences was observed (Guzik et al., 2013). However, due to the limited amount of information in this case, further studies are needed to determine HRA role as a clinically significant predictor.

In Kaczmarek et al. (2019), participant’s response to stimuli that were applied to induce positive emotions higher number of heart rate decelerations was noted. HRV analysis has been successfully applied in psychophysiology research on emotions (Lane et al., 2009). Therefore, it would be worth to examine the role of HRA in emotional states further.

Further research to investigate the change of HRA during different phases of HUTT would be advised among people of different ages and genders. We hope that short-term HRA study may be beneficial in testing patients with diseases in which HUTT is a diagnostic tool, e.g., vasovagal syncope.

### Limitations

Our study limitations include disregarding the effect of person’s weight. Extending the time of ECG recordings would improve research results.

## Conclusion

Our study confirms that response of the cardiovascular system to tilt during HUTT may impact upon human heart rate, HRV, and HRA. The change of RR interval length is statistically significant, likewise the change of short- and long-term variability parameters—SD1 and SD2. Short-term HRA index results (GI) were significantly different in supine and tilt phases of HUTT. We did not observe statistically significant change of PI and GI_LT_. Statistically significant increase of long monotonic RR series was observed. We assume that short-term HRA analysis and monotonic RR series length analysis are the methods that may qualify the phases of the HUTT. We hope that HRA analysis during tilt test is an introduction to the assessment of the diagnostic value of HRA measurements in people with vasovagal syndrome and chronic fatigue syndrome. We see potential of HRA analysis as a tool for more accurate diagnosis of people suffering from diseases related to disorders of the nervous system which are frequently difficult to determine and even to distinguish. Constant development of computational methods allowing for more precise estimation of HRA for healthy individuals is in progress.

## Data Availability Statement

The datasets presented in this article will be made available on request after adequate justification. Requests to access the datasets should be directed to PZ, p.zalewski@cm.umk.pl.

## Ethics Statement

The studies involving human participants were reviewed and approved by the Ethics Committee, Ludwik Rydygier Memorial Collegium Medicum in Bydgoszcz, Nicolaus Copernicus University, Toruń. The participants provided their written informed consent to participate in this study.

## Author Contributions

RP, KB, and PZ: conceptualization and methodology. RP: software, formal analysis, data curation, visualization, and writing—original draft preparation. RP, KB, and PZ: validation and investigation. PZ: resources. KB, PZ, JN, and SK: writing—review and editing. KB: supervision. KB and PZ: project administration and funding acquisition. All authors read and agreed to the published version of the manuscript.

## Conflict of Interest

The authors declare that the research was conducted in the absence of any commercial or financial relationships that could be construed as a potential conflict of interest.

## Footnotes

1 https://github.com/jaropis/hrvhra

2 www.r-project.org
